# A New Eudesmane Sesquiterpene Glucoside from *Liriope muscari *Fibrous Roots

**DOI:** 10.3390/molecules16119017

**Published:** 2011-10-26

**Authors:** Hai Ming Zhang, Gang Li Wang, Chun Qi Bai, Peng Liu, Zi Mu Liu, Qi Zhi Liu, Yong Yan Wang, Zhi Long Liu, Shu Shan Du, Zhi Wei Deng

**Affiliations:** 1 State Key Laboratory of Earth Surface Processes and Resource Ecology, Beijing Normal University, Beijing 100875, China; 2 National Institutes for Food and Drug Control, Beijing 100050, China; 3 Department of Entomology, China Agricultural University, Haidian District, Beijing 100193, China; 4 Analytical and Testing Center, Beijing Normal University, Beijing 100875, China

**Keywords:** *Liriope muscari*, Nematocidal activity, *Bursaphelenchus xylophilus*, 1,4-epoxy-*cis*-eudesm-6-*O*-*β*-D-glucopyranoside

## Abstract

The screening of several Chinese medicinal herbs for nematocidal properties showed that the ethanol extract of *Liriope muscari *fibrous roots possessed significant nematocidal activity against the pine wood nematode (*Bursaphelenchus xylophilus*). From the ethanol extract, a new constituent (1,4-epoxy-*cis*-eudesm-6-*O*-*β*-D-glucopyranoside) and three known glycosides [1*β*,6*α*-dihydroxy-*cis*-eudesm-3-ene-6-*O*-*β*-D-glucopyranoside (liriopeoside A), 1*β*,6*β*-dihydroxy-*cis*-eudesm-3-ene-6-*O*-*β*-D-glucopyranoside, and 1*α*,6*β*-dihydroxy-5,10-*bis*-*epi*-eudesm-4(15)-ene-6-*O*-*β*-D-glucopyranoside] were isolated by bioassay-guided fractionation. The structures were elucidated by 1D and 2D NMR and MS techniques. 1,4-Epoxy-*cis*-eudesm-6-*O*-*β*-D-glucopyranoside possessed moderate nemato-cidal activity against *B. xylophilus* with a LC_50 _value of 339.76 μg/mL, while liriopeoside A (LC_50_ = 82.84 μg/mL) and 1*β*,6*β*-dihydroxy-*cis*-eudesm-3-ene-6-*O*-*β*-D-glucopyranoside (LC_50_ = 153.39 μg/mL) also exhibited nematocidal activity against *B. xylophilus*. The crude extract of *L. muscari* fibrous roots exhibited nematocidal activity against the pine wood nematode with a LC_50_ value of 182.56 μg/mL.

## 1. Introduction

**Figure 1:**
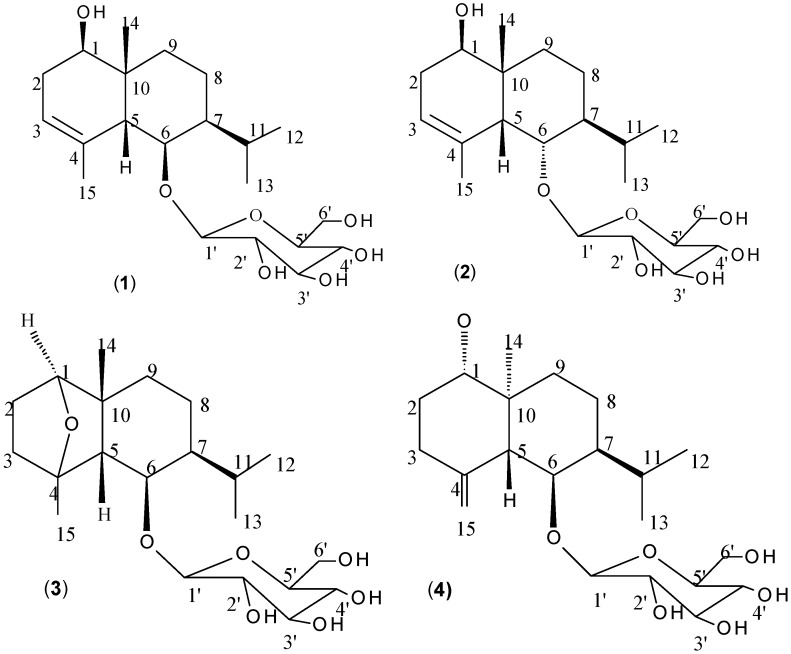
Structures of compounds isolated from *Liriope muscari *fibrous roots.

*Liriope muscari* (Decaisne) L.H. Bailey (Family: Liliaceae), is a species of low, herbaceous flowering plant from East Asia (China, Japan and Korea) occurring in shady forests at elevations of 330 to 4,600 feet. It is a perennial with grass-like evergreen foliage and lilac-purple flowers which produce single seeded berries on a spike in the fall [1]. The roots of this species are used as a substitute for Radix Ophiopogonis (*Ophiopogon japonicus*) in some areas of China [2,3]. Its roots have been employed in Traditional Chinese Medicine as an expectorant, antitussive, tonic agent and also used to treat various inflammation-related diseases such as pharyngitis, bronchitis, pneumonia, cough in addition to showing pharmacological effects on the cardiovascular system [3,4]. Previous phytochemical investigations revealed that *L. muscari* mainly contained amides, steroids, steroidal glycosides (saponins), triterpenoids, flavonoids and essential oil [5,6,7,8,9,10,11]. The essential oil of *L. muscari* had fumigant toxicity against the rice weevil (*Sitophilus oryzae*) but no strong toxicity was found [12]. However, during our screening program for new agrochemicals from local wild plants and Chinese medicinal herbs, the ethanol extract of *L. muscari* fibrous roots was found to possess nematocidal activity against the pine wood nematode [*Bursaphelenchus xylophilus* (Steiner and Buherer) Nickle]. We report here the isolation and characterization of a new eudesmane sesquiterpene glucoside, 1,4-epoxy-*cis*-eudesm-6-*O*-*β*-D-glucopyranoside (**3**) and three known glycosides (Figure 1) by bioassay-guided fractionation and their nematocidal assessment against *B. xylophilus*. Liriopeoside A (**1**, 1β,6β-dihydroxy-*cis*-eudesm-3-ene-6-*O*-*β*-D-glucopyranoside) was first isolated from *L. muscari *[9] and 1α,6β-dihydroxy-*cis*-eudesm-3-ene-6-*O*-*β*-D-glucopyranoside (**2**) and 1α,6β-dihydroxy-5,10-bis-epi-eudesm-4(15)-ene-6-*O*-*β*-D-glucopyranoside (**4**) were isolated from *Aster scaber *[15] and *Parepigynum funingense* [16], respectively.

## 2. Results and Discussion

### 2.1. Chemistry

1,4-Epoxy-*cis*-eudesm-6-*O*-*β*-D-glucopyranoside (**3**, Figure 1) was obtained as a colorless powder from the ethanol extract of *L. muscari* fibrous roots. The ^1^H-NMR spectrum of compound **3** led to the identification of the following representative signals: two methyl singlet protons at d 1.40 and 1.06, two methyl doublet signal protons at δ 0.89 (*d*, *J* = 6.0 Hz) and 0.87 (*d*, *J *= 6.0 Hz), and an anomeric proton at δ 4.19 (*d*, *J* = 7.7 Hz). In the ^13^C-NMR (including DEPT) spectrum, 21 carbon signals appeared, which included four methyl carbons at δ_C_ = 31.2, 22.4, 22.3, and 20.7; five methylene carbons at δ_C_ = 61.7, 30.1, 29.4, 25.9 and 20.0; three oxygenated methine carbons at δ_C_ = 87.7, 85.5, and 76.2; three methine carbons at δ_C_ = 56.7, 43.1, and 26.6 and six signals assignable to the glucose moiety (δ_C_ = 102.8, 77.4, 77.2, 74.5, 70.7 and 61.7).

**Figure 2:**
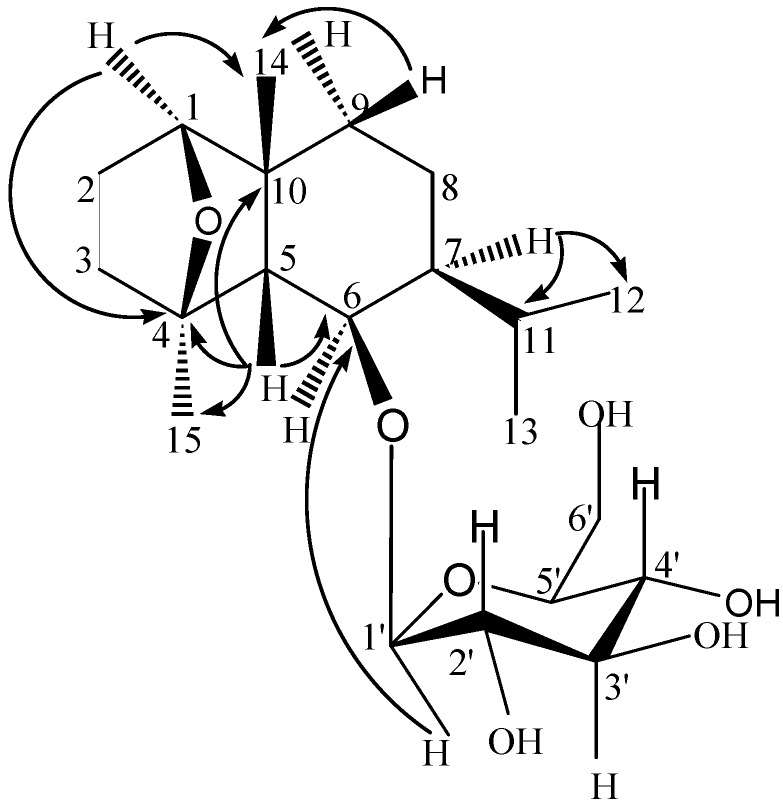
Key HMBC correlations of compound **3**.

The NMR data of compound **3** were similar to those of 1*β*,4*β*,6*β*-trihydroxy-*cis*-eudesmane-6-*O*-*β*-D-glucopyranoside isolated from *O. japonicus* [9]. The major difference in compound **3** was the downfield chemical shifts for the carbons at C-l (δ_C_ = 85.5) and C-4 (δ_C_ = 87.7) in contrast to C-l (δ_C_ = 74.8) and C-4 (δ_C_ = 71.5) in compound 1*β*,4*β*,6*β*-trihydroxy-*cis*-eudesmane-6-*O*-*β*-D-glucopyranoside, uggesting that there was an epoxy bridge between C-1 and C-4, a fact confirmed by a key HMBC correlation between H-1 (δ_H_ = 3.74, *t*, *J* = 3.1 Hz) and C-4 (δ_C_ = 87.7) (Figure 2). Furthermore, the molecular formula of 1*β*,4*β*,6*β*-trihydroxy-*cis*-eudesmane-6-*O*-*β*-D-glucopyranoside was determined to be C_21_H_3__8_O, whereas the molecular formula of compound **3 **was determined to be C_21_H_36_O_7_, suggesting a loss of a water molecule (18 amu).

The HSQC and HMBC spectrum indicated that δ 3.74 (H-1) has connectivity with δ 31.2 (C-14), 87.7 (C-4) and 56.7 (C-5); δ 1.66 (H-5) has connectivity with δ 31.2 (C-14), 42.7 (C-10), 20.7 (C-15), 87.7 (C-4) and 76.2 (C-6); δ 1.40 (H-7) has connectivity with δ 26.6 (C-11) and 22.4 (C-12).

The coupling constant (*J* = 7.7 Hz) of the anomeric proton at δ_H_ = 4.19 indicated the D-glucose moiety was in the *β*-form. The glycosidic position was established by HMBC, with a long-range correlation observed between H-1′ (δ_H_ = 4.19, *d*, *J* = 7.7 Hz) and C-6 (δ_C_ = 76.2).

The relative configuration of compound **3** was deduced by comparing its ^13^C-NMR spectral data and coupling constants with those of 1*β*,4*β*,6*β*-trihydroxy-*cis*-eudesmane-6-*O*-*β*-D-glucopyranoside. The chemical shifts of C-5, C-6, C-7, C-10 and C-15 in compound **3** and those in 1*β*,4*β*,6*β*-trihydroxy-*cis*-eudesmane-6-*O*-*β*-D-glucopyranoside were similar, indicating that the H-6 and H-7 were *α*-oriented, the methyl group at C-4 was *α*-substituted. The coupling constant of H-1 (δ_H_ = 4.24, *t*, *J* = 3.2 Hz) for compound **3** was quite similar to that of 1*β*,4*β*,6*β*-trihydroxy-*cis*-eudesmane-6-*O*-*β*-D-glucopyranoside (δ_H_ = 3.68, *t*, *J* = 3.1 Hz), thus the configuration of H-1 was *α*-oriented. The proposed structure of compound **3** was in accordance with ^1^H-^1^H COSY, HSQC, and HMBC spectra. Therefore, the structure of compound **3** was established as 1,4-epoxy-*cis*-eudesm-6-*O*-*β*-D-glucopyranoside.

### 2.2. Biological Activity

The ethanol extract of *L. muscari* fibrous roots exhibited toxicity against the pine wood nematode (*B. xylophilus*) with an LC_50_ value of 182.56 μg/mL (Table 1).

**Table 1:** Nematocidal activity of ethanol extract of *Liriope muscari *fibrous roots and isolated constituents against *B. xylophilus*.

Treatments	Concentrations (μg/mL)	72 h LC_50_ (μg/mL)	95% Fiducial limits	Chi-Square Tests (χ^2^)
Ethanol extract	50.00–800.00	182.56	19.79–24.46	9.45
**1**	50.00–800.00	82.84	76.80–93.49	1.95
**2**	50.00–800.00	153.39	140.44–169.46	5.83
**3**	50.00–800.00	339.76	302.99–366.65	3.97
**4**	50.00–800.00	465.68	435.88–508.33	2.28
Carbofuran	1.50–50.00	3.76	2.62–4.99	3.78

1*β*,6*β*-Dihydroxy-*cis*-eudesm-3-ene-6-*O*-*β*-D-glucopyranoside showed weak toxicity against *B. xylophilus*, with an LC_50_ value of 339.76 μg/mL. However, the other two isolated compounds, 1*β*,6*α*-dihydroxy-*cis*-eudesm-3-ene-6-*O*-*β*-D-glucopyranoside and 1,4-epoxy-*cis*-eudesm-6-*O*-*β*-D-gluco-pyranoside exhibited higher nematocidal activity against *B. xylophilus*, with LC_50_ values of 82.84 μg/mL and 153.39 μg/mL, respectively. Compared to the positive control, carbofuran (LC_50_ = 3.76 μg/mL), the most toxic compound was 22 times less activite against the pine wood nematode.

## 3. Experimental

### 3.1. General

Melting points were measured on a Buchi 535 apparatus and were uncorrected. High-resolution mass spectra were determined on a Bruker micrOTOF-Q spectrometer, equipped with Apollo II electrospray ionization source with ion funnel, operated in the positive ion mode. ^1^H- and ^13^C-NMR spectra were recorded on Bruker Avance DRX 500 instrument using DMSO-d_6_ as a solvent with TMS as internal standard.

### 3.2. Plant Material

Fresh fibrous roots of *L. muscari* were collected from Quanzhou City (24.54° N latitude and 118.37° E longitude), Fujian Province, China in May 2010. The species was identified by Dr. Liu. Q.R. (College of Life Sciences, Beijing Normal University), and the voucher specimens (BNU-CMH-Dushuahan-2010-05-24-012 were deposited at the Herbarium (BNU) of College of Life Sciences, Beijing Normal University. The roots were air-dried and ground to a powder using a grinding mill (Retsch Muhle, Germany).

### 3.3. Extraction and Isolation of Active Ingredients

The powder (5 kg) was extracted with 80% ethanol (10 L) at room temperature over a period of 2 weeks. The extracts were concentrated to afford a syrup (1.8 Kg), which was dissolved in water (1,800 mL) and chromatographed on a AB-8 resin (Nankai University, Tianjin, China) column (120 mm in diameter and 1.0 m in height) eluting with a gradient of EtOH-H_2_O (0:100, 10: 90, 50: 50, 90: 10) to give four fractions. The 90% ethanol eluent was concentrated under vacuum to obtain the crude glycosides (51 g) which were dissolved in water (500 mL), and then fractionated with *n*-BuOH (500 mL × 5) to yield the *n*-BuOH-soluble extract (45 g) after evaporation of the solvent. The *n*-BuOH-soluble extract was subjected to silica gel column chromatography (900 g, 160-200 mesh, Qingdao Marine Chemical Plant, Shandong Province, China), using a CHCl_3_-MeOH solvent system of increasing polarity (from 100% CHCl_3__, _CHCl_3_:MeOH = 100:2, CHCl_3_:MeOH = 100:5, … 100% MeOH) to afford 60 fractions. Fractions 17–20 (2.0 g, elution with CHCl_3_-MeOH = 100:10) were combined according to their TLC similarity (precoated silica gel GF_254_ plates, Yantai Jiangyou Silica Gel Development Co. Ltd., Shandong, China) and separated by ODS (40 g, Agela Technologies, Tianjin, China) column chromatography to give five subfractions. The subfractions were purified by semi-preparative HPLC [Waters 2695-2996 liquid chromatograph, Agilent ZORBAX Eclipse XDB-C_18_ columns (250 mm × 4.6 mm and 250 mm × 9.4 mm i.d.)] to yield compound **1** (55 mg) using MeOH-H_2_O (35:65) as mobile phase at a flow rate of 3 mL/min, compound **2** (60 mg) using MeCN-H_2_O (22:78) as mobile phase at a flow rate of 3 mL/min, compound **3** (50 mg) using MeOH-H_2_O (25:75) as mobile phase at a flow rate of 3 mL/min and compound **4** (42 mg) using MeOH-H_2_O (40:60) as mobile phase at a flow rate of 3 mL/min.

### 3.5. Compound Characterization

*1β,6β-Dihydroxy-cis-eudesm-3-ene-6-O-β-D-glucopyranoside* (**1**). White powder, HR-ESIMS *m/z*: 423.2356 [M + Na]^+^. C_21_H_36_O_7_. ^1^H-NMR (DMSO-d_6_, 500 MHz) δ ppm: 4.24 (1H, t, *J* = 3.2 Hz, H-1), 2.26 (1H, d, *J* = 15 Hz, H-2), 1.87 (1H, m, H-2), 5.19 (1H, br, s, H-3), 2.54 (1H, s, H-5), 4.21 (1H, t, *J* = 2.6 Hz, H-6), 0.70 (1H, m, H-7), 1.49 (1H, m, H-8), 1.41 (1H, m, H-8), 1.24 (1H, m, H-9), 1.03 (1H, m, H-9), 1.79 (1H, m, H-11), 0.85 (3H, d, *J* = 6.8 Hz, H-12), 0.91 (3H, d, *J* = 6.5 Hz, H-13), 1.10 (3H, s, H-14), 1.64 (3H, s, H-15), 4.23 (1H, d, *J* = 7.7 Hz, H-1′). ^13^C-NMR (DMSO-d_6_, 125 MHz) δ ppm: 133.6 (C-4), 120.1 (C-3), 105.3 (C-1’), 77.5 (C-5’), 77.3 (C-6), 77.1 (C-1), 74.9 (C-3’), 73.8 (C-2’), 71.1 (C-4’), 61.9 (C-6’), 46.2 (C-5), 45.4 (C-7), 36.1 (C-10), 32.2 (C-9), 31.7 (C-2), 27.5 (C-11), 25.0 (C-14), 22.4 (C-15), 22.1 (C-13), 21.4 (C-12), 20.2 (C-8). The ^1^H- and ^13^C-NMR (DMSO-d_6_) spectral data are consistent with published data [9].

*1α,6β-Dihydroxy-cis-eudesm-3-ene-6-O-β-D-glucopyranoside* (**2**). White powder, HR-ESIMS *m/z*: 423.1462 [M + Na]^+^. C_21_H_36_O_7_. ^1^H-NMR (DMSO-d_6_, 500 MHz) δ ppm: 3.90 (1H, t, *J* = 3.2 Hz, H-1), 5.21 (1H, br.s, H-3), 2.01 (1H, m, H-5), 4.38 (1H, br.s, H-6), 1.50 (1H, m, H-7), 1.78 (1H, m, H-8), 1.64 (1H, m, H-8), 1.97 (1H, m, H-9), 1.59 (1H, m, H-9), 1.90 (1H, m, H-11), 4.25 (1H, d, *J* = 7.0 Hz, H-1’), 1.00 (3H, d, *J* = 6.0 Hz, H-12), 0.95 (3H, d, *J* = 6.5 Hz, H-13), 0.86 (3H, s, H-14), 1.79 (3H, s, H-15). ^13^C-NMR (DMSO-d_6_, 125 MHz) δ ppm: 135.9 (C-4), 120.4 (C-3), 102.5 (C-1’), 77.6 (C-5’), 78.2 (C-3’), 78.8 (C-1), 71.7 (C-6), 75.4 (C-2’), 71.8 (C-4’), 61.9 (C-6’), 45.1 (C-7), 46.7 (C-5), 37.6 (C-10), 34.8 (C-9), 31.7 (C-2), 30.0 (C-11), 22.2 (C-13), 22.1 (C-12), 12.7 (C-14), 21.2 (C-8), 22.1 (C-15). The ^1^H- and ^13^C-NMR (DMSO-d_6_) spectral data are consistent with published data [15].

*1,4-Epoxy-cis-eudesm-6-O-β-D-glucopyranoside* (**3**). White powder, mp 215.0–217.6 °C, HR-ESIMS *m/z*: 423.3416 [M + Na]^+^. C_21_H_36_O_7_. ^1^H-NMR (DMSO-d_6_, 500MHz) δ ppm: 3.74 (1H, t, *J* = 3.1 Hz, H-1), 1.51 (1H, m, H-2), 1.36 (1H, m, H-2), 1.85 (1H, m, H-3), 1.09 (1H, m, H-3), 1.66 (1H, d, *J* = 5.0 Hz, H-5), 3.78 (1H, d, *J* = 2.0 Hz, H-6), 1.40 (1H, m, H-7), 1.86 (1H, m, H-8), 1.48 (1H, m, H-8), 1.49 (1H, m, H-9), 1.19 (1H, m, H-9), 2.10 (1H, m, H-11), 0.89 (3H, d, *J* = 6.0 Hz, H-12), 0.87 (3H, d, *J* = 6.0 Hz, H-13), 1.06 (3H, s, H-14), 1.40 (3H, s, H-15), 4.19 (1H, d, *J* = 7.7 Hz, H-1’), 3.12 (1H, t, *J* = 7.7 Hz, H-2’), 3.07 (1H, m, H-3’), 2.92 (1H, t, *J* = 8.0 Hz, H-4’), 3.45 (1H, t, *J* = 7.8 Hz, H-5’), 3.68 (1H, dd, *J* = 12.0, 7.7 Hz, H-6’), 3.05 (1H, m, H-6’). ^13^C-NMR (DMSO-d_6_, 125MHz) δ ppm: 102.8 (C-1’), 87.7 (C-4), 85.5 (C-1), 77.4 (C-5’), 77.2 (C-3’), 76.2 (C-6), 74.5 (C-2’), 70.7 (C-4’), 61.7 (C-6’), 56.7 (C-5), 43.1 (C-7), 42.7 (C-10), 31.2 (C-14), 30.1 (C-3), 29.4 (C-9), 26.6 (C-11), 25.9 (C-8), 22.4 (C-12), 22.3 (C-13), 20.7 (C-15), 20.0 (C-2).

*1α,6β-Dihydroxy-5,10-bis-epi-eudesm-4(15)-ene-6-O-β-D-glucopyranoside* (**4**). White powder, HR-ESIMS *m/z*: 423.2210 [M + Na]^+^. C_21_H_36_O_7_. ^1^H-NMR (DMSO-d_6_, 500MHz) δ ppm: 2.25 (1H, br.s, H-11), 1.07 (3H, d, *J* = 6.4 Hz, H-12), 0.98 (3H, d, *J* = 6.4 Hz, H-13), 0.99 (3H, s, H-14), 5.64 (1H, br.s, H-15), 4.82 (1H, br.s, H-15), 3.89 (1H, m, H-1), 4.61 (1H, d, *J* = 7.7 Hz, H-1’). ^13^C-NMR (DMSO-d_6_, 125MHz) δ ppm: 146.9 (C-4), 108.3 (C-15), 100.4 (C-1’), 77.3 (C-1), 76.5 (C-5’), 75.6 (C-3’), 74.9 (C-2’), 71.1 (C-6), 70.7 (C-4’), 61.8 (C-6’), 53.8 (C-5), 46.8 (C-7), 40.5 (C-3), 34.4 (C-10), 31.4 (C-9), 29.4 (C-2), 29.2 (C-11), 21.5 (C-14), 21.0 (C-13), 20.2 (C-8),20.0 (C-12).The ^1^H- and ^13^C-NMR (DMSO-d_6_) spectral data are consistent with published data [16].

### 3.2. Nematocidal Assay

The pine wood nematode (*B. xylophilus*) was isolated from chips of infested pine wood collected in Taizhou city (28.41° N latitude and 121.27° E longitude), Zhejiang Province, China and extracted by Baermann funnel techniques [13]. The pine wood nematode isolate was rinsed three times with sterile distilled water and reared on a lawn of *Botrytis cinerea* cultured on potato dextrose agar medium in the dark at 25 °C. Petri-dishes (9 cm in diameter) with fully grown fungus were inoculated with *B. xylophilus* and left until fungal mycelia were completely consumed (3–5 days). The cultured nematodes (mixed stage) were separated from fungal cultures by centrifuging at 8,500 rpm at 20 °C, rinsed with sterile distilled water and collected. An aqueous suspension of the nematodes (ca. 5,000 nematodes per mL) was prepared by appropriate dilution for use as a working stock.

Range-finding studies were run to determine the appropriate testing concentrations. A serial dilution of the ethanol extract (pure compounds, five concentrations) was prepared. The crude extract/compounds were first dissolved in ethanol and the final concentration of the ethanol solution was 2%. The *in vitro* tests were performed in wells of 12-well plates. Solution containing test compounds/extract (20 μL) and dilution with about 100 nematodes (20 μL) were added to each well and the final volume of each vial was 1 mL. The plates were incubated in an incubator at 25 °C. Dead and active nematodes were recorded in an interval of 24 h for 72 h using a microscope (×20). The nematodes were considered dead if they gave no response to physical stimuli such as mechanical stirring and pricking with the point of a needle. Six replicates were performed for each treatment. A 2% alcohol in H_2_O solution was used as a negative control and carbofuran as a positive control. Results from all replicates for the pure compounds and ethanol extract were subjected to probit analysis using the PriProbit Program V1.6.3 to determine LC_50_ values and their 95% confidence intervals [14].

## 4. Conclusions

Based on mass screening of medicinal herbs, the ethanol extract of *L. muscari* fibrous roots was found to possess toxicity against the pine wood nematode (*B. xylophilus*). A novel and three known eudesmane sesquiterpene glycosides were isolated and identified from the ethanol extract of *L. muscari *by bioactivity-guided fractionation. The four isolated constituents and the crude extract exhibited nematocidal activity against the pine-wood nematode (*B. xylophilus*).

## Supplementary Materials

Supplementary materials can be accessed at: http://www.mdpi.com/1420-3049/16/11/9017/s1.
